# Unraveling bulk degradation mechanisms of wide-bandgap perovskite absorbers for tandem applications

**DOI:** 10.1039/d5el00199d

**Published:** 2026-02-26

**Authors:** Chiara Ongaro, Mostafa Othman, Christophe Ballif, Christian M. Wolff, Aïcha Hessler-Wyser

**Affiliations:** a École Polytechnique Fédérale de Lausanne (EPFL), Institute of Electrical and Micro Engineering (IEM), Photovoltaics and Thin-Film Electronics Laboratory (PV-lab) Rue de la Maladière 71b Neuchâtel 2000 Switzerland chiara.ongaro@epfl.ch; b CSEM, Sustainable Energy Center Rue Jaquet-Droz 1 Neuchâtel 2000 Switzerland

## Abstract

Wide-bandgap (WBG) perovskite absorbers play a pivotal role in enabling high-efficiency tandem solar cells; yet, their long-term operational stability remains a significant hurdle to commercialization. Although interface engineering has led to promising progress, these improvements have not yet translated into the level of stability required for market readiness. Recent studies increasingly highlight the intrinsic instability of the perovskite bulk as a key limiting factor. This review examines the underlying mechanisms that compromise bulk stability in WBG perovskites (1.65–1.8 eV), covering both mixed-cation mixed-halide absorbers and fully inorganic systems such as CsPbI_3_. Particular attention is given to the intrinsic factors that compromise the long-term stability of WBG perovskites, including compositional inhomogeneities, crystallization-driven disorder, insufficient crystallinity and texture, nanoscale phase impurities, and intrinsic phase-instability phenomena. Mixed-cation mixed-halide formulations, widely used to access tandem-relevant bandgaps, frequently exhibit spatially uneven elemental distributions and light- or thermally induced halide segregation, both of which introduce structural and electronic disorder. In parallel, inorganic WBG absorbers such as CsPbI_3_ are predominantly limited by their strong propensity for converting to non-perovskite phases. In both material families, the crystallization pathway critically dictates the spatial distribution of components and the incorporation of defects. The resulting heterogeneities, together with nanoscale impurities and secondary phases, serve as initiation sites for absorber degradation under operational conditions. This review discusses emerging strategies aimed at overcoming these challenges, including compositional engineering, crystallization control, and targeted passivation. By addressing the root causes of bulk instability, this work outlines guidance toward achieving the long-term stability required for WBG perovskites in tandem photovoltaic technologies.

## Abstract 2

Wide-bandgap (WBG) perovskite absorbers play a pivotal role in enabling high-efficiency tandem solar cells; yet, their long-term operational stability remains a significant hurdle to commercialization.

## Graphical abstract

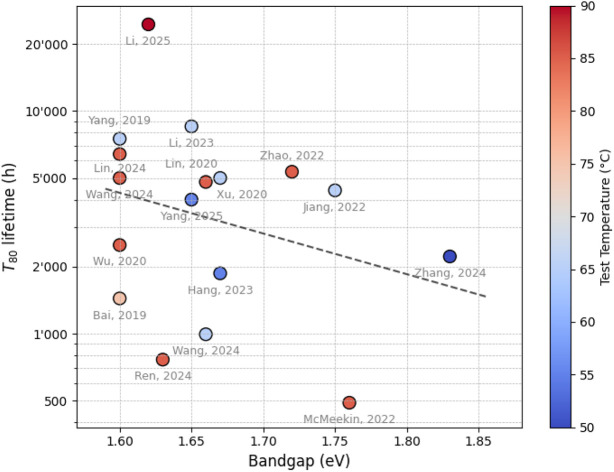

**Broader context**

Wide-bandgap (WBG) perovskite absorbers (*E*_g_ > 1.65 eV) are central to enabling perovskite/silicon tandem solar cells, which have already achieved record efficiencies approaching 35%. Their bandgap tunability makes them uniquely suited for multi-junction applications, yet long-term stability remains the key bottleneck preventing their commercialization. In contrast to lower-bandgap perovskites, for which relatively stable devices have been demonstrated, reports of robust WBG devices remain scarce—particularly under realistic operating conditions involving continuous illumination and thermal stress. While interface engineering has led to incremental gains in efficiency and stability, these advances remain insufficient for practical deployment. Increasingly, intrinsic bulk instabilities—arising from compositional heterogeneities, complex crystallization pathways, phase segregation, and nanoscale impurities—are being recognized as the dominant factors limiting WBG device longevity. This review consolidates recent insights into these bulk-related degradation mechanisms and evaluates emerging strategies to mitigate them, from compositional engineering to crystallization control and defect passivation. By clarifying why stable WBG devices remain elusive and highlighting promising directions for overcoming these challenges, this work outlines a roadmap toward the material quality required for durable tandem photovoltaics.

## 1 Introduction

The rapid development and remarkable efficiency gains demonstrated by perovskite solar cells (PSCs) over the past decade have positioned them as front-runners among emerging photovoltaic (PV) technologies.^1–6^ Single-junction PSCs have achieved record power conversion efficiencies (PCEs) of 27% on small-area devices (0.06 cm^2^), closely approaching the performance of state-of-the-art silicon heterojunction solar cells, which currently reach 27.3% PCE on industrial-scale modules (243 cm^2^) and are nearing their theoretical performance ceiling of 29.51%.^7–12^ Similarly, previous theoretical analyses have estimated the efficiency potential for optimized single-junction PSCs to exceed 28%.^13^ A compelling avenue to surpass the inherent limitations of single-junction devices involves their integration into multijunction configurations. Among these, perovskite/silicon tandem solar cells (PSTs) have attracted significant interest, primarily due to their compatibility with established silicon technologies.^14^ Recent advancements have propelled certified efficiencies of PSTs to 34.9% for 1 cm^2^ devices, underscoring their potential for enhanced performances.^11,15^ However, realizing the commercial viability of PSTs requires addressing intrinsic stability challenges inherent to perovskite absorbers, falling short of the stability standards set by silicon devices.^16–20^

In PSTs, the top perovskite cell ideally employs a wide-bandgap (WBG) absorber, typically in the range of 1.65–1.7 eV, to optimize solar spectrum utilization and minimize thermalization losses.^14,21,22^ Mixed-cation mixed-halide compositions have emerged as promising candidates for this role, and the most efficient PSTs reported to date rely on such formulations.^23,24^ However, achieving these wider bandgaps through compositional engineering introduces additional stability challenges compared to their lower-bandgap (1.5–1.6 eV) counterparts. In particular, the incorporation of mixed halide systems (iodide, I^−^, bromide, Br^−^ and chloride, Cl^−^) and mixed A-site cations-formamidinium (FA^+^), cesium (Cs^+^), methylammonium (MA^+^), as well as pseudohalide anions, is essential for tuning the perovskite bandgap to values suitable for tandem solar cells. These mixed compositions introduce complex crystallization dynamics, leading to increased structural, compositional, and electronic disorder, which ultimately compromises the long-term operational stability of the absorber layer.^25–27^ Alongside these mixed-cation mixed-halide compositions, the all-inorganic perovskite CsPbI_3_ also offers a tandem-relevant bandgap and a compositionally simpler framework. Yet, despite its chemical simplicity, CsPbI_3_ suffers from its own intrinsic phase-instability challenges, most notably the black-to-yellow transition driven by lattice strain, defect chemistry, and thermodynamic factors.^28,29^

**Fig. 1:**
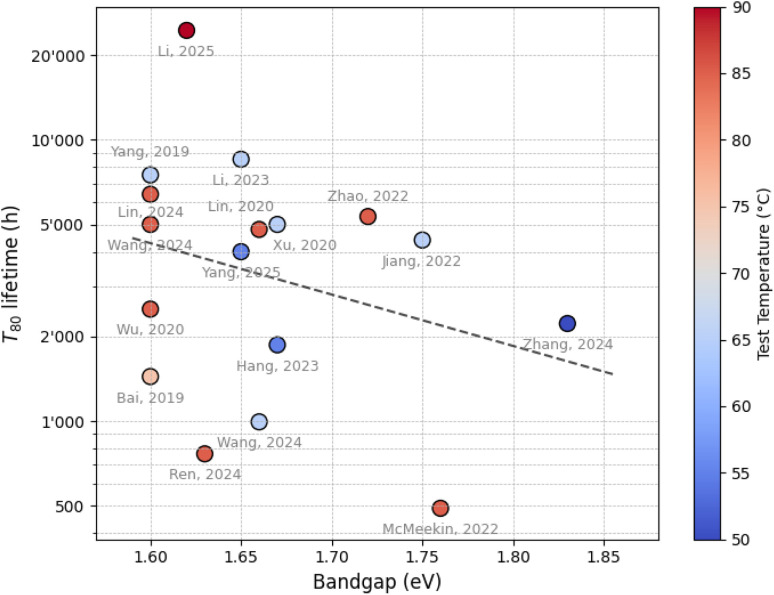
*T*_80_ lifetime of WBG absorbers as a function of bandgap energy and testing temperature, illustrating stability trends under accelerated aging. Each data point and its corresponding reference are listed in Table S1 of the SI.

While several studies have been dedicated to enhancing the stability of lower-bandgap organic–inorganic perovskite devices, robust operational stability for WBG perovskite solar cells remains comparatively underexplored.^30–33^ This gap is especially evident under accelerated stress conditions involving prolonged continuous light-soaking at elevated temperatures.^34^Fig. 1 provides an overview of recent stability data for WBG absorbers, illustrating *T*_80_ lifetimes as a function of bandgap and testing temperature. A tabulated summary of the data points, along with relevant experimental details such as bandgap, stability conditions, device architecture, size of active area, and corresponding references, is provided in the SI, Table S1.

To tackle the limited operational stability of WBG perovskite absorbers, significant research efforts have focused on engineering the interfaces of WBG perovskite devices.^35–40^ These strategies, ranging from interface passivation to the use of more stable charge transport layers, have led to meaningful, though still incremental, improvements in both efficiency and stability. Despite these advances, current stability levels remain insufficient for long-term operation in tandem architectures.^41–43^ Increasing attention has therefore shifted toward the intrinsic quality of the perovskite bulk material, which is now recognised as a central factor limiting durability.^44,45^ A recent review by Jiang *et al.*^42^ provided a broad device-level perspective on WBG perovskite stability, emphasising degradation under external stressors such as illumination, heat, humidity, and the role of encapsulation. In contrast, the present work focuses specifically on the intrinsic bulk-instability mechanisms that govern the long-term behaviour of WBG absorbers relevant to PSTs, including mixed-cation mixed-halide formulations and the inorganic CsPbI_3_ system, which, as mentioned, currently constitute the most promising and widely adopted compositions for tandem photovoltaic applications. Specifically, we review and critically examine how compositional heterogeneities, complex crystallisation pathways, structural defects, nanoscale impurities, and phase-instability phenomena collectively impact the long-term stability of these absorbers, and we further discuss emerging approaches aimed at mitigating these intrinsic degradation pathways.

## 2 Compositional heterogeneity

Mixed-halide PSCs often exhibit local compositional heterogeneities, stemming from uneven distribution of both A-site cations and X-site halides across the perovskite lattice^37,41,46–50^ These inhomogeneities pose a significant challenge in fabricating high-quality solution-processed thin films, ultimately restricting their potential for use in efficient multi-junction solar cells.^49,51^ In that regard, maintaining compositional homogeneity is believed to play a role in supporting the structural stability of metal halide perovskites, which may in turn influence the long-term operational stability of the devices.^52,53^ While most studies on phase segregation in mixed cation-halide perovskites focus on film aging to investigate cation and anion migration under external stressors, there is increasing evidence that such segregation is initially present in the freshly formed films. These intrinsic inhomogeneities not only compromise stability at the outset but may also serve as seeds that exacerbate degradation when exposed to external factors.^54^ The following sections explore some of the underlying possible causes of these inhomogeneities and assess their direct impact on the intrinsic film's instability.

### 2.1 Halide compositional heterogeneity

WBG mixed-halide PSCs typically exhibit halide heterogeneity, manifesting as localized regions of halide enrichment or depletion.^50,55–57^ One of the factors contributing to such compositional inhomogeneities could be the difference in solubility among halide precursors in common processing solvents, such as dimethylformamide (DMF) or dimethyl sulfoxide (DMSO). Specifically, Br precursors are generally less soluble compared to iodide counterparts, leading to preferential crystallization behavior and halide segregation during film formation.^50,57,58^ Br^−^, being less soluble than I^−^, precipitates earlier during crystallization, leading to the initial formation of Br-rich domains.^58–60^ In conventional antisolvent-assisted solution-processed cells, these Br-rich phases preferentially crystallize at the perovskite–air interface, in which the evaporation of the solvent is triggered, resulting in a compositional gradient where Br^−^ is concentrated near the top of the film.^46,51^ Given that these chemical variations originate from the intrinsic differences between Br and I ions, and become more pronounced with higher Br content, a widely adopted strategy to mitigate halide inhomogeneities and phase segregation to limit the Br concentration below 20%.^61^ For instance, the composition Cs_0.25_FA_0.75_Pb(Br_0.2_I_0.8_)_3_, with a bandgap of ≈1.68 eV, has been shown to remain stable against halide segregation under 10 minutes of illumination at 10 suns.^25^ However, this approach inherently constrains the possibility of exploring higher bandgaps, which may be desirable for other multijunction architectures beyond PSTs.

**Fig. 2:**
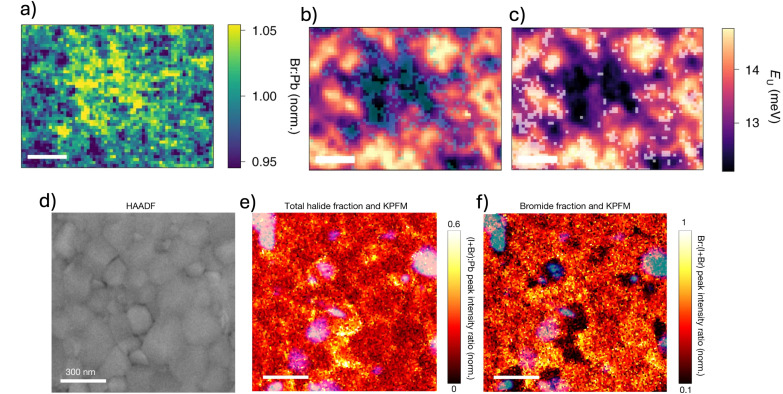
(a–c) Characterization of perovskite thin films with composition (Cs_0.05_FA_0.78_MA_0.17_)Pb(I_0.83_Br_0.17_)_3_. (a) Normalized elemental map showing the Br : Pb ratio across the film. (b and c) Maps of Urbach energy, overlaid with regions containing the highest (>80th percentile, light blue) and lowest (<20th percentile, light grey) bromine content, respectively. Reprinted from Frohna *et al.*^62^ Copyright 2021, Springer Nature. (d–f) Structural and compositional analysis of the same perovskite films. (d) HAADF-STEM image highlighting grain morphology. (e) Ratio of total halide counts (*I*(I Lα) + *I*(Br Kα)) relative to lead intensity (*I*(Pb Lα)), derived from STEM-EDX measurements; some grains and boundaries exhibit halide enrichment. (f) Bromine content represented as a fraction of total halide counts, *I*(Br Kα)/(*I*(I Lα) + *I*(Br Kα)). Notably, grains enriched in total halides appear deficient in bromide, while the surrounding matrix shows a more uniform Br distribution. In both (e) and (f), compositional data were normalized between 0 and 1 using min–max scaling based on the respective elemental intensity maps. Blue overlays in (b) and (c) represent trap-rich areas identified *via* KPFM, typically located at interfaces between compositionally distinct grains and the more uniform background material. Reprinted from Doherty *et al.*^64^ Copyright 2020, Springer Nature.

These local spatial chemical inhomogeneities can also significantly impact the optoelectronic properties of perovskite films by introducing structural instabilities and bandgap fluctuations. Such variations create energy barriers that hinder efficient carrier transport, disrupt the local chemical potential, and lead to non-uniform defect distributions.^51,62,63^ For instance, Frohna *et al.* demonstrated the direct impact of halide inhomogeneities on optoelectronic properties using nano-X-ray fluorescence (n-XRF) mapping.^62^ By mapping the Br : Pb ratio in as-deposited films (before light exposure), they revealed significant spatial variations in halide composition (Fig. 2a). Correlating these maps with Urbach energy measurements, Fig. 2b and c, they found that regions with higher Br^−^ content exhibited lower electronic disorder, suggesting that halide distribution plays a crucial role in determining the material's local defect density and charge transport efficiency. Doherty *et al.* found that nanoscale trap clusters are primarily located at the boundaries between compositionally inhomogeneous grains and the compositionally uniform surrounding material, highlighting the strong link between halide inhomogeneities and defect formation, particularly at grain boundaries.^64^ Using high-angle annular dark field scanning transmission electron microscopy (HAADF-STEM), scanning transmission electron microscopy with energy-dispersive X-ray spectroscopy (STEM-EDX), and kelvin probe force microscopy (KPFM) on the same scan area of a mixed-cation mixed-halide perovskite film, they demonstrated that local halide fluctuations contribute to charge-trapping sites, ultimately impacting optoelectronic performance (Fig. 2d–f).^64^

Finally, chemical inhomogeneities have been reported to exacerbate light-induced halide segregation,^47,49,65,66^ which has been proposed as a contributing factor to open-circuit voltage (*V*_oc_) losses and operational degradation in WBG PSCs.^67–69^ Yet, the behavior of this segregation remains an active point of debate: some studies observe that photo-induced I/Br demixing can relax once illumination is removed, indicating a largely reversible process under moderate excitation conditions,^66,70^ whereas other reports show that under prolonged illumination, elevated temperatures, or in the presence of higher defect densities, halide redistribution can become persistent and, in certain cases, effectively irreversible.^71–74^ At the same time, the origin of *V*_oc_ losses is itself not fully resolved, with several works pointing to interfacial or contact-related recombination pathways as dominant contributors.^68,75^ Nevertheless, achieving a uniform halide distribution is generally considered beneficial, leading to enhanced performance and stability of devices.

### 2.2 A-site cations compositional heterogeneity

**Fig. 3:**
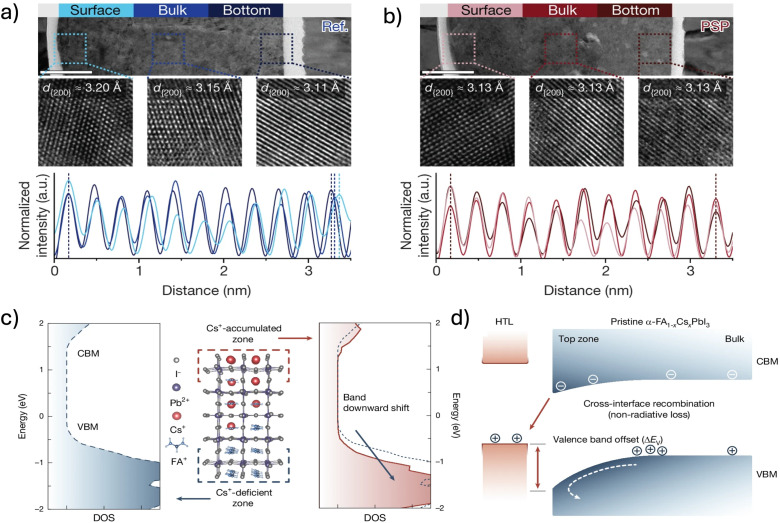
(a and b) TEM cross-sectional images of (a) reference films and (b) samples treated with 1-(phenylsulfonyl)pyrrole to promote A-site compositional uniformity. Reprinted from Liang *et al.*^48^ Copyright 2023, Springer Nature. (c) Calculated DOS for the Cs^+^-rich region (red solid line) and Cs^+^-deficient region (blue dashed line) in pristine α-FA_1−*x*_Cs_*x*_PbI_3_ perovskites. The slab model used in the calculation is depicted in the center. (d) Schematic illustration of band misalignment induced by Cs^+^ accumulation at the surface, as confirmed by UPS depth profiling. CBM: conduction band minimum; VBM: valence band maximum. Reprinted from Li *et al.*^76^ Copyright 2024, Springer Nature.

A-site cation segregation, much like halide segregation, presents a significant challenge in mixed-cation perovskites, where Cs^+^ and FA^+^ exhibit intrinsic phase segregation. Local variations in Cs^+^ concentration disrupt compositional uniformity, leading to lattice distortions, structural mismatches, and potentially contributing to the emergence of photo-inactive secondary phases, all of which undermine perovskite film stability and performance.^54,55,63,76–78^ Liang *et al.* provided direct evidence of these compositional inhomogeneities using cross-sectional transmission electron microscopy (TEM), revealing structural discontinuities at distinct depths within the perovskite films (bottom, bulk, and top, Fig. 3a). These inhomogeneities were effectively mitigated upon the inclusion of a suitable additive (Fig. 3b).^48^ Their findings indicate a preferential accumulation of Cs^+^ at the bottom of the film, inferred from a measurable decrease in lattice spacing, further corroborated by depth profiling *via* time-of-flight secondary ion mass spectrometry (ToF-SIMS), which confirmed the vertical distribution of Cs^+^ within the perovskite layer (Fig. 6a). Further analysis demonstrated that these A-site compositional discontinuities induce shifts in both the conduction and valence bands in regions with high Cs^+^ concentration (Fig. 3c).^76^ The resulting band misalignment (Fig. 3d) leads to quasi Fermi level splitting (QFLS) limitations and increases interfacial contact resistance, thereby reducing charge extraction efficiency and exacerbating non-radiative recombination losses. Together, these factors significantly degrade device performance and undermine long-term stability, emphasizing the need for strategies that regulate A-site cation incorporation to minimize segregation effects.

One possible explanation for this A-site cation segregation lies in the soft base nature of Cs^+^, which interacts more strongly with PbI_3_^−^ than FA^+^, leading to preferential crystallization of Cs^+^-rich domains during early film growth.^48^ Additionally, the lower solubility of Cs compared to FA^+^ might promote its early precipitation at the precursor stage, accelerating crystallization and reinforcing chemical heterogeneities within the film.^47^

## 3 Crystallization engineering for compositional control

Crystallization plays a pivotal role in dictating the final distribution of halides and A-site cations in perovskite thin films. Since compositional inhomogeneities are often inherited from the as-formed film, controlling nucleation, crystal-growth kinetics, and phase formation is critical for ensuring compositional uniformity. The following subsections explore strategies developed to achieve homogeneous halide and cation distributions *via* crystallization engineering.

### 3.1 Homogenizing halide-mixing *via* crystallization engineering

**Fig. 4:**
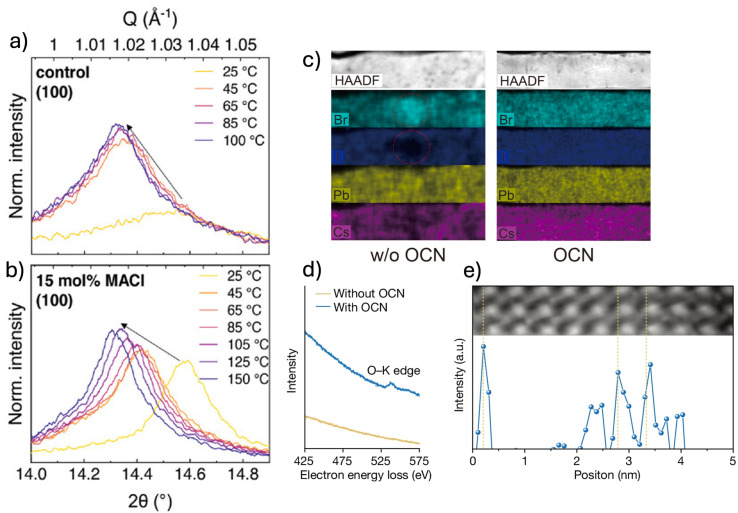
(a and b) XRD patterns showing the evolution of the cubic perovskite (100) peak during step-wise annealing of FA_0.83_Cs_0.17_Pb(I_0.60_Br_0.40_)_3_ films prepared with 15 mol% MACl, compared with control samples processed without MACl. The control film was annealed at 100 °C in a N_2_ glovebox, while MACl-containing films were annealed at 150 °C under ambient air (relative humidity ≈30%). Reprinted from Shen *et al.*^79^ Copyright 2023, Wiley-VCH GmbH. (c) EDX elemental maps of Cs, Pb, I, and Br^−^ in mixed-cation mixed-halide perovskite films incorporating 5% OCN, based on a nominal FA_0.60_MA_0.15_Cs_0.25_Pb(I_0.45_Br_0.50_OCN_0.05_)_3_ composition. Red dashed circles highlight Br^−^ and I^−^ inhomogeneity in films processed without OCN. (d) EELS spectra of the O–K edge comparing films with and without 5% OCN. (e) Atomic-resolution EELS line scan of the O–K edge, showing oxygen atoms localised near the edges of Pb atomic columns. Reprinted from Liu *et al.*^83^ Copyright 2024, Springer Nature.

A widely adopted strategy to mitigate halide inhomogeneities in PSCs is Cl^−^ alloying, which enables the maintenance of low Br^−^ content while achieving the desired bandgap, concurrently improving halide distribution uniformity. Shen *et al.* demonstrated that incorporating methylammonium chloride (MACl) into the precursor solution introduces a third halide species (Cl^−^), which has been shown to suppress halide segregation during crystallization.^79^ This effect is primarily driven by the formation of a Cl-rich intermediate phase at the early stages of crystallization. *In situ* X-ray diffraction (XRD) measurements (Fig. 4a and b) revealed a stronger cubic (100) reflection shifted toward higher angles upon MACl addition compared to control samples, indicative of a temporary lattice contraction associated with Cl^−^ incorporation. Upon thermal annealing, the peak shifts back to lower angles, likely indicating the gradual substitution of Cl^−^ by I^−^ and Br^−^. These findings are further supported by *in situ* grazing-incidence wide-angle X-ray scattering (GIWAXS) measurements and photoluminescence (PL) analysis under different thermal treatments, confirming the role of Cl^−^ in regulating halide incorporation. This preferential nucleation of chloride-containing phases is attributed to the stronger interaction between Pb^2+^ and Cl^−^, as supported by potential of mean force (PMF) calculations, which show a higher binding affinity of Pb^2+^ for Cl^−^ compared to Br^−^ and I^−^. By delaying the early precipitation of Br^−^, this mechanism prevents the premature formation of Br-rich domains, ensuring a more uniform halide distribution throughout the perovskite lattice upon annealing.

Instead of substituting Br^−^ with another halide, such as Cl^−^, some studies have also explored the incorporation of pseudohalides to mitigate halide inhomogeneities. Thiocyanate (SCN^−^) and cyanate ions have been found to be particularly suitable for this purpose, showing to effectively substitute for traditional halides.^80–82^ Notably, Liu *et al.* showed the successful integration of cyanate (OCN^−^), which has a similar ionic size to Br^−^, into WBG perovskite lattices.^83^ Through density functional theory (DFT) calculations and high-resolution electron energy loss spectroscopy (EELS) measurements, they demonstrated that cyanate anions predominantly localize near Pb atoms within the perovskite lattice, indicating that OCN^−^ substitutes for halide ions (Fig. 4d and e). This substitution improves halide distribution uniformity (Fig. 4c), which the authors attribute to modifications in the crystallization process that enable the concurrent incorporation of both Br^−^ and I^−^ ions.

**Fig. 5:**
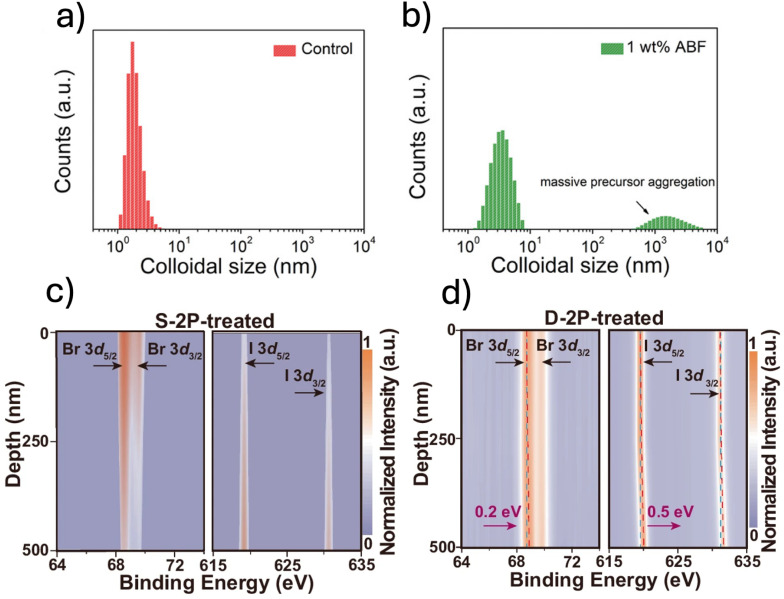
(a and b) DLS spectra of Rb_0.05_Cs_0.05_(FA_0.83_MA_0.17_)Pb(I_0.83_Br_0.17_)_3_ perovskite precursor solutions (a) without and (b) with the ABF additive. Reprinted from Zheng *et al.*^47^ Copyright 2023, Wiley-VCH GmbH. (c) Depth-resolved XPS spectra showing Br 3d and I 3d core-level signals in S-2P-treated FA_0.80_Cs_0.15_MA_0.05_Pb(I_0.70_Br_0.30_)_3_ perovskite films. (d) Depth-profiling XPS analysis of I 3d and Br 3d signals in the same absorber. Reprinted from Wang *et al.*^37^ Copyright 2024, Springer Nature.

Following a similar rationale to Cl^−^-alloying, other additives have been reported to influence the nucleation and crystallization processes of perovskite films.^84^ For instance, the multifunctional additive 4-(2-aminoethyl)benzenesulfonyl fluoride (ABF) has been shown to significantly alter crystallization dynamics, homogenizing vertical halide distribution.^47^ Through Fourier-transform infrared spectroscopy (FTIR) and nuclear magnetic resonance (NMR) measurements, it was found that ABF strongly interacts with the perovskite precursors, suggesting the formation of pre-nucleation clusters at the film surface during the initial stages of grain growth. This is further supported by dynamic light scattering (DLS) measurements (Fig. 5a and b), which show a notable increase in precursor colloid size in the presence of ABF. These clusters likely serve as growth templates for the crystallization of underlying layers, promoting uniform halide distribution throughout the film. Similarly to Cl^−^ alloying, such pre-nucleation clusters could function as an intermediate crystallization phase, effectively lowering the nucleation energy barrier,^47^ and enabling simultaneous nucleation of different halide species, preventing the preferential crystallization of Br-rich domains.

Another approach which has been suggested to enhance the halogen phase distribution is to modify the buried p-type interface in PSC. Wang *et al.* introduced a double-layer self-assembled monolayer (SAM) of 2-(9*H*-carbazol-9-yl)ethyl phosphonic acid (2-PACz) on NiO_*x*_, demonstrating its ability to regulate halide crystallization dynamics.^37^ The exposed phosphate groups in the SAM interact with [PbX_6_]^4−^ octahedra (X = I^−^, Br^−^) *via* hydrogen bonding, serving as nucleation sites that facilitate more uniform halide incorporation during perovskite film formation. This effect was confirmed by depth-profiling X-ray photoelectron spectroscopy (XPS) measurements, which revealed a more homogeneous halogen distribution in treated films (Fig. 5c and d).

The common denominator among these approaches is the regulation of crystallization dynamics to ensure simultaneous incorporation of halide species and prevent the abrupt Br-rich phase formation. Whether through Cl^−^ alloying, pseudohalide substitution, precursor additives, or interface modifications, these strategies rely on modifying Pb^2+^ coordination to influence halide binding affinities and precursor interactions. By slowing or guiding nucleation and growth, they counteract bromide's natural tendency to precipitate first, thereby stabilizing halide distribution across the perovskite lattice. These findings underscore that controlling crystallization kinetics can be a thermodynamic lever to achieve homogeneous halide compositions and improve device stability.

### 3.2 Homogenizing A-site cation mixing *via* crystallization engineering

**Fig. 6:**
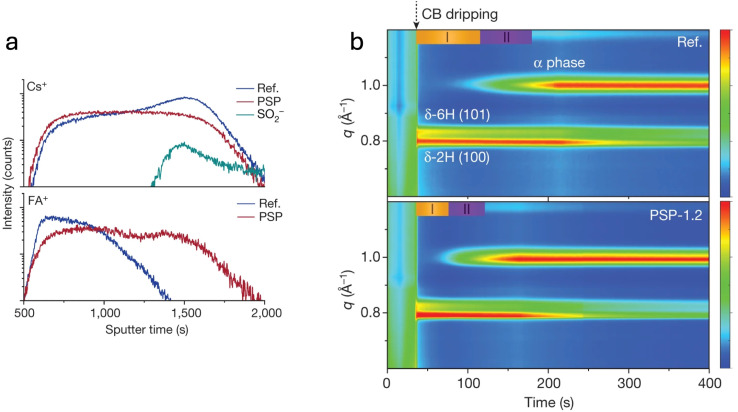
(a) ToF-SIMS depth profiles showing cation distribution in reference (blue) and PSP-treated (red) FA_0.95_Cs_0.05_PbI_3_ perovskite devices. (b) *In situ* GIWAXS patterns of the reference and treated films, capturing the crystallization dynamics (period I) and the subsequent phase transition (period II). Color scales are normalized between 0 and 1. Reprinted from Liang *et al.*^48^ Copyright 2023, Springer Nature.

To address the issue of uniform A-site cation distribution, crystallization engineering has been explored as a strategy to regulate A-site cation incorporation and suppress phase segregation. Liang *et al.* tackled this challenge by introducing 1-(phenylsulfonyl) pyrrole (PSP) additive, which effectively suppresses Cs^+^/FA^+^ segregation, leading to a more uniform cation distribution in the final films (Fig. 6a).^48^*In situ* GIWAXS measurements reveal that PSP significantly influenced both crystallization dynamics and the phase transition from the hexagonal δ-phase to the cubic α-phase perovskite (Fig. 6b). By comparing reference and PSP-treated films, they observed that the additive accelerates crystallization (Period I)—from the moment of chlorobenzene (CB) dripping until the appearance of the α-phase—as well as the phase transition and stabilization of the α-phase (Period II). Further insights from extended X-ray absorption fine structure (EXAFS) and NMR measurements suggested that PSP coordinates with Pb atoms *via* its sulfone functional group, thereby regulating the crystallization dynamics of Cs^+^ and FA^+^. This interaction reduces their tendency to segregate, promoting a more homogeneous perovskite film, which ultimately results in enhanced stability compared to the chemically inhomogeneous reference films.

**Fig. 7:**
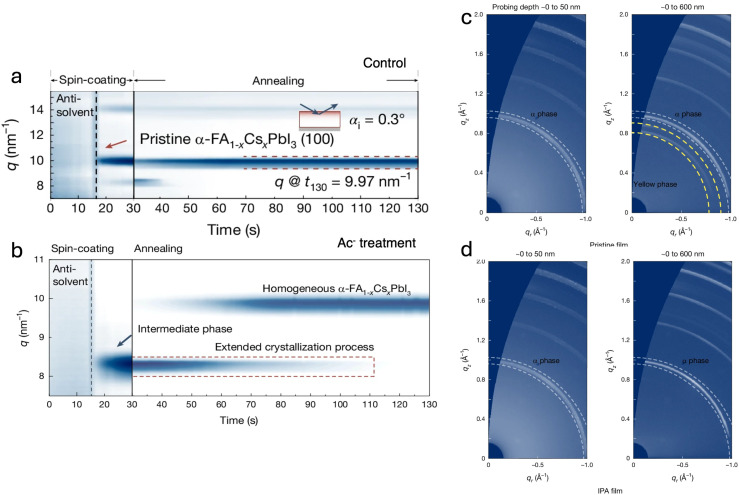
(a) Time-resolved GIWAXS spectra of pristine α-FA_1−*x*_Cs_*x*_PbI_3_ films. (b) Time-resolved GIWAXS spectra of acetate-treated α-FA_1−*x*_Cs_*x*_PbI_3_ films. (c and d) Two-dimensional GIWAXS scattering patterns of aged α-FA_0.94_Cs_0.06_PbI_3_ films collected at different probing depths for (c) pristine and (d) acetate-treated samples. Reprinted from Li *et al.*^76^ Copyright 2024, Springer Nature.

Another effective strategy to enhance A-site cation homogeneity involves the incorporation of acetate ions (Ac^−^) as surface ligands in the precursor solution.^76^ This modification has been found to promote an alternative crystallization pathway *via* the formation of the intermediate δ-FA_1−*x*_Cs_*x*_PbI_3_ phase, which has been shown to exhibit a lower Cs^+^ diffusion barrier, thereby facilitating cation homogenization. Interestingly, unlike the approach of Liang *et al.*, where the additive accelerates the crystallization process compared to the reference, the formation of this intermediate phase in the acetate-modified system actually slows it down.^48^ Particularly, *in situ* GIWAXS measurements revealed the clear presence of the δ-phase at *q* ≈ 8.34 nm^−1^ (Fig. 7b), with the emergence of the (100) α-phase occurring only during the annealing step, rather than immediately after antisolvent dripping, as for the reference film (Fig. 7a). Notably, the presence of this intermediate δ-phase, enhanced with the Ac^−^ treatment, was also evident in the films of Liang *et al.* (Fig. 6b). Devices based on FA_0.94_Cs_0.06_PbI_3_ (1.5 eV) with enhanced out-of-plane cation uniformity demonstrated significantly improved stability, achieving *T*_95_ = 2000 h under continuous illumination at 85 °C and 60% relative humidity. This improved stability has been attributed to the absence of yellow-phase formation at the bottom of aged films, as evidenced by depth-dependent GIWAXS measurements (Fig. 7c and d). The presence of this phase in pristine films has been linked to Cs-deficient regions, which trigger local phase instability over time. These findings highlight the crucial role of maintaining a homogeneous A-site cation distribution in suppressing phase decomposition and extending device longevity.

## 4 Phase instability of inorganic WBG perovskites

An alternative route to achieve WBG absorbers suitable for PSTs applications, while circumventing the challenges associated with compositional heterogeneities in mixed-cation and mixed-halide systems is to employ intrinsically simpler compositions. In this context, the all-inorganic cesium lead iodide (CsPbI_3_) perovskite, containing a single A-site cation (Cs^+^) and a single halide (I^−^), has emerged as a promising candidate. With a bandgap of approximately 1.73 eV, CsPbI_3_ falls within the optimal range for use as the top absorber in PST solar cells, combining favorable optoelectronic properties with the potential for enhanced thermal stability due to the absence of volatile organic components.^85^

**Fig. 8:**
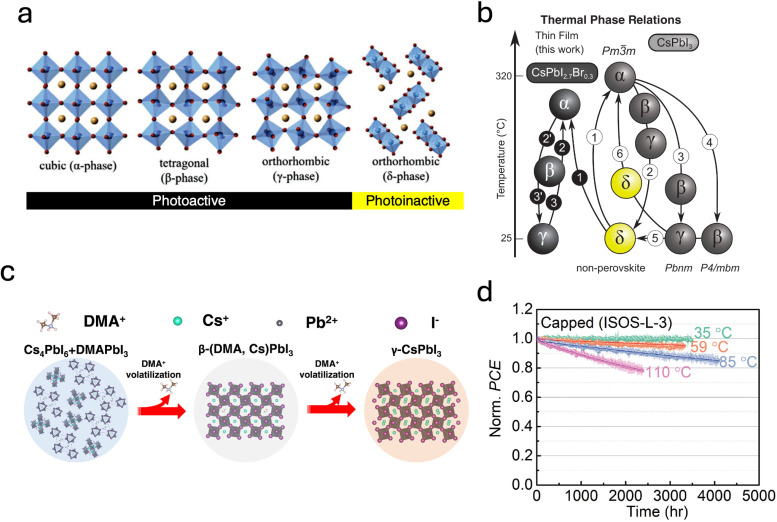
(a) Crystalline phases of CsPbI_3_, image adapted from Qin *et al.*^91^ Copyright 2021, Wiley-VCH GmbH. (b) Thermal phase transitions of CsPbI_3_ in comparison with the phase behavior of strained CsPbI_2.7_Br_0.3_. Reprinted from Steele *et al.*^92^ Copyright 2021, The American Association for the Advancement of Science. (c) Structural evolution of CsPbI_3_-based thin films during their formation when processed with the DMA^+^ organic cation. Reprinted from Jiang *et al.*^93^ Copyright 2023 Elsevier Inc. (d) MPPT tracking of CsPbI_3_ capped PSCs operating at 35°, 59 °C, 85 °C, and 110 °C under continuous full-spectrum illumination. Reprinted from Zhao *et al.*^94^ Copyright 2022, The American Association for the Advancement of Science.

At the same time, CsPbI_3_ presents its own intrinsic stability challenges. Structurally, CsPbI_3_ can crystallize in four distinct phases (Fig. 8a): cubic (α), tetragonal (β), and two orthorhombic forms, the photoactive black γ-phase and the photoinactive yellow δ-phase.^29^ At room temperature, the δ-phase is thermodynamically favored, as the small A-site cation size results in a Goldschmidt tolerance factor (*t*) below 0.8, outside the range typically required for perovskite stability (0.8 to 1.0).^86^ Heating the yellow δ-phase above 320 °C induces its conversion to the black α-phase; however, upon cooling, the system reverts sequentially through the β and γ phases before returning to the δ-phase, as illustrated in Fig. 8b.

The fundamental driving forces behind this phase instability have been the focus of extensive investigation. Early lattice-dynamics calculations by Marronnier *et al.*^29^ revealed that CsPbI_3_ possesses the intrinsically soft lattice characteristic of lead-halide perovskites. This softness manifests as strongly anharmonic lattice vibrations and soft phonon modes, which promote octahedral tilting and lower the energy barrier for transformation into the non-perovskite δ-phase. These findings align with the experimental work of Li *et al.*,^86^ who demonstrated that the stability of the black perovskite phase in CsPbI_3_ and related alloys is governed by geometric constraints, where a low tolerance factor enhances octahedral tilting and lattice distortion, ultimately favoring the formation of the δ-phase.

Beyond these thermodynamic considerations, more recent theoretical and experimental studies have highlighted the crucial role of surface defects in facilitating this phase transition. Combined theoretical modeling by Guo *et al.* and experimental observations by Wylie *et al.* demonstrated that surface iodide vacancies act as nucleation centers for δ-phase growth.^87,88^ These vacancies induce local lattice distortions and octahedral tilting that drive the perovskite framework toward the orthorhombic δ-structure. Once nucleated, the transformation propagates through the lattice in a domino-like fashion, as emerging δ-domains further strain adjacent regions and promote additional vacancy formation.^87^ Moisture exposure exacerbates this process: solvation of surface halides increases vacancy concentration and provides further nucleation sites for the δ-phase, thereby accelerating degradation of the black perovskite phase.^29,89^ This behavior confirms the well-known moisture sensitivity of CsPbI_3_, which markedly accelerates its transition to the non-photoactive δ-phase.

The phase instability of CsPbI_3_, whose microscopic origins are yet to be fully uncovered, remains a major bottleneck for its reliable integration into photovoltaic devices. Consequently, the development of effective stabilization strategies for the dark perovskite phases has become a focal point of current research, particularly those that avoid high-temperature processing, which can be incompatible with PSTs and can degrade self-assembled monolayers typically used as hole-selective layers in p–i–n architectures.^90^

### 4.1 Stabilization strategies for dark-CsPbI_3_ perovskite

A variety of strategies have been developed to stabilize the dark phases of CsPbI_3_, targeting either the cubic α-phase, the tetragonal β phase or orthorhombic γ one. The main approaches include ionic doping and alloying at the A-, B-, and X-sites, the use of additives, surface functionalization, and strain engineering.

Compositional tuning at the A, B, and X sites has been widely explored as a route to stabilise dark-phase CsPbI_3_ with the purpose of modulating the perovskite tolerance factor and reduce the propensity for octahedral distortion. At the A site, partial substitution of Cs^+^ with small alkali cations such as K^+^,^95^ Rb^+^,^96^ or Na^+^,^97^ or with larger organic ions such as MA^+^ or FA^+^, has been shown to modulate the perovskite tolerance factor and reduce the propensity for octahedral distortion. Similarly, B-site alloying with ions including Mn^2+^,^98^ Ca^2+^,^99^ Bi^3+^,^100^ or Sn^2+^^101^ can further enhance lattice coherence and suppress defect formation, while X-site halide mixing with Br^−^ or Cl^−^ can improve structural stability, albeit at the cost of bandgap widening when used excessively.^102–105^ The work of Steele *et al.* have indicated that many of these stabilising dopants also act by reducing the spontaneous lattice strains that promote octahedral tilting in CsPbI_3_, thereby favouring higher-symmetry black phases and slowing their conversion to the non-perovskite δ-phase.^28^ However, in practice, these compositional adjustments provide only partial stabilization of the dark phase, and reported devices generally show limited efficiencies and modest operational stability, suggesting the need for more effective and durable stabilization strategies.

The most widely adopted strategy to stabilise dark-phase CsPbI_3_, and the one underlying many of the highest-performing inorganic perovskite devices reported to date, relies on introducing hydroiodic acid (HI) into CsPbI_3_ precursor solutions prepared in dimethylformamide (DMF).^85^ This route enables formation of the black phase at relatively low temperatures (around 100 °C). Ke *et al.* clarified that HI reacts with DMF to generate dimethylammonium iodide (DMAI) *in situ*, which modifies crystallisation and facilitates dark-phase formation.^106^ The precise role of the organic cation DMA^+^ in this process, however, remains a matter of active debate. Wang *et al.* argued that DMAI behaves primarily as a volatile intermediate that templates crystal growth without being incorporated into the final structure.^107^ In contrast, Marshall *et al.* provided evidence that DMA^+^ can substitute Cs^+^ at the A site, forming Cs_1−*x*_DMA_*x*_PbI_3_ alloys.^108^ More recently, Jiang *et al.* provided further insight into these differing observations by showing that the incorporation and retention of DMA^+^ in CsPbI_3_ are highly sensitive to processing temperature: low-temperature annealing preserves a small amount of DMA^+^ and yields a more symmetric tetragonal β-phase, whereas higher temperatures promote DMA^+^ volatilisation and result in the fully inorganic orthorhombic γ-phase, as depicted in Fig. 8c.^93^ Although the crystallisation pathways remain complex, DMAI-assisted processing consistently enhances the humidity resistance, film quality, device performance, and operational stability of CsPbI_3_ compared to pristine films, making it, so far, one of the most impactful strategies for stabilising dark-phase CsPbI_3_ absorbers.

Beyond DMAI based routes, other additives have also been explored to stabilize dark phase CsPbI_3_. Notably, the incorporation of poly(vinylpyrrolidone) (PVP) into CsPbI_3_ precursors was showed to markedly suppresses the transition to the yellow phase by coordinating surface Cs ions.^94,109,110^ A particularly significant advance was reported by Zhao *et al.*, who combined PVP additive engineering with an inorganic Cs_2_PbI_2_Cl_2_ capping layer to suppress both bulk and interfacial degradation pathways.^94,111^ This approach yielded some of the best stability under thermal stress achieved for WBG perovskite devices, with encapsulated devices enduring continuous operation at 110 °C for over 2100 hours before reaching *T*_80_,^94^Fig. 8d. Beyond inorganic capping layers, surface functionalization through organic 2D layers, such as those formed by tetra-FPDMA^112^ or by bulky ammonium salts including PEAI^113^ or PEABr,^114^ has similarly been shown to kinetically hinder the transition to the yellow δ-phase by passivating surface defects and stabilizing the perovskite lattice.

Finally, strain engineering has also been explored as a means of assisting the stabilization of dark-phase CsPbI_3_. Studies have shown that substrate-induced biaxial strain can kinetically trap the black γ-phase at room temperature,^92^ and that external pressure or lattice-mismatch strain can similarly retard the transition toward the δ-phase in nanocrystals.^115,116^

## 5 Crystallographic orientation and film texturing

The crystallization dynamic of perovskite films inherently leads to domains characterized by distinct facet orientations, each presenting unique atomic structures and local chemical environments. Such facet-specific variations significantly impact electronic, physical, and chemical properties.^117–122^ These differences can result in facet-dependent degradation behaviors, where less stable orientations degrade faster, accelerating the overall deterioration of the perovskite film.^123^ Beyond facet orientation, achieving high crystallinity and enhanced film texture has also proven critical for stabilizing WBG perovskites. Films with strong texturing tend to exhibit fewer structural defects, which enhances their resistance to degradation and extends device operational lifetimes.^123,124^

Ma *et al.* were the first to provide direct evidence linking crystallographic facet orientation to stability in perovskite films, demonstrating significant facet-dependent degradation behaviors in formamidinium lead iodide (FAPbI_3_) absorbers.^123^ Specifically, the authors showed that the (100) facet exhibited notably higher susceptibility to moisture-induced degradation, driven by strong water adsorption that elongated Pb–I bonds and accelerated the transition to the non-photoactive δ-phase. In contrast, the (111) facet demonstrated enhanced structural robustness, effectively resisting such hydration-induced phase transformations. In a separate study, Gao *et al.* similarly found that perovskite films exposing the (111) facet exhibited superior resistance to moisture, heat, and light stress compared to their (001)-oriented counterparts.^125^ Taken together, these independent investigations underscore the enhanced intrinsic stability of the (111) facet in FAPbI_3_-based absorbers.

**Fig. 9:**
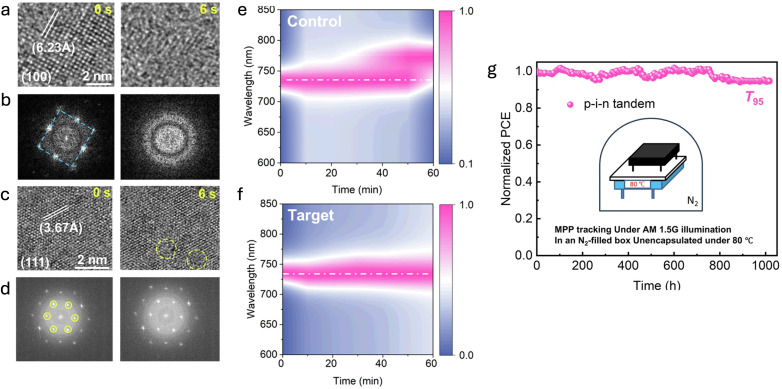
(a) and (b) Time-sequential HRTEM images showing the structural evolution of the (100) plane under continuous electron-beam exposure, together with the corresponding FFT images. (c) Structural evolution of the (111) plane under identical conditions, with the associated FFT images shown in (d). (e) and (f) Evolution of cathodoluminescence (CL) spectra under electron-beam irradiation for control (e) and target (f) films. (g) Long-term MPP tracking of 1 cm^2^ p–i–n tandem devices incorporating (111)-oriented WBG FA/MA/Cs–Pb(I_0.7_Br_0.3_)_3_ perovskite, measured at 80 °C, showing *T*_95_ ≈ 1000 h. Reprinted from Yao *et al.*^126^ Copyright 2025, Springer Nature.

Similarly, extending this investigation to WBG perovskites, Yao *et al.* recently explored the role of crystallographic orientation on the stability of perovskite films with a bandgap of approximately 1.68 eV.^126^ Their study revealed significant facet-dependent stability under illumination and electron-beam exposure, highlighting that the (111) facet exhibits superior resistance to degradation compared to the (100) facet. Through *in situ* HRTEM, they demonstrated enhanced electron-beam stability of (111)-oriented grains, which retained their structural integrity under prolonged beam exposure, unlike the rapidly degraded (100) grains (Fig. 9a–d). While the precise degradation mechanisms under electron-beam conditions remain complex and not yet fully understood—potentially involving decomposition of organic cations—the authors observed a higher activation energy for ion migration on the (111) facet, which may also contribute to its reduced susceptibility to beam- and light-induced degradation. Furthermore, by incorporating trioctylphosphine oxide (TOPO) into the precursor solution, the authors successfully induced preferential (111) facet growth, significantly suppressing halide segregation, as shown from their time-dependent cathodoluminescence (CL) spectroscopy experiment, Fig. 9e and f. The authors demonstrated remarkable operational stability for perovskite/silicon tandem devices achieving a *T*_80_ lifetime exceeding 1000 hours at the maximum power point (MPP) at 80 °C, Fig. 9g.

**Fig. 10:**
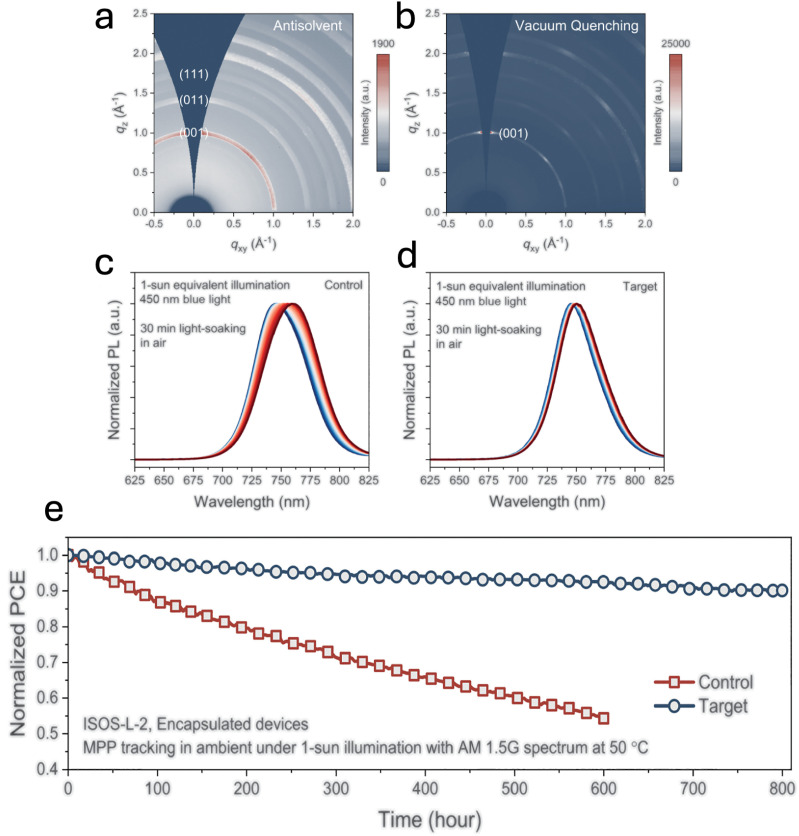
(a) and (b) GIWAXS patterns of FA_0.78_Cs_0.22_Pb(I_0.82_Br_0.18_)_3_ absorbers prepared with OAmI, processed *via* the antisolvent (a) and vacuum-quenching (b) methods. (c) and (d) *In situ* PL measurements of control (c) and target (d) WBG films under 450 nm LED illumination (intensity corrected to 1-sun conditions). (e) MPP tracking of encapsulated control and target PST devices under continuous 1-sun AM 1.5G illumination at 50 °C (ISOS-L-2). Reprinted from Chen *et al.*^127^ Copyright 2024, The American Association for the Advancement of Science.

Optimizing the texture, crystallinity and grain size of WBG perovskite films have also emerged as an essential strategies for improving operational stability. Recently, Chen *et al.* reported significantly enhanced crystal texturing in mixed-cation mixed-halide WBG absorbers by adopting vacuum- or gas-quenching deposition methods instead of traditional antisolvent-based techniques.^127^ This finding aligns closely with previous work by Jiang *et al.*, who similarly showed improved crystal orientation and stability in WBG perovskite solar cells achieved *via* gas-quenching methods.^46^ Furthermore, Chen *et al.* introduced oleylammonium iodide (OAmI) as an additive to selectively promote nucleation of the cubic perovskite phase, thereby suppressing the formation of undesirable secondary phases during crystallisation.^127^ GIWAXS measurements (Fig. 10a and b) demonstrated that films processed by vacuum or gas quenching in the presence of this additive exhibited substantially improved crystallinity and stronger preferential (001) orientation compared to films prepared with the conventional antisolvent method. This enhanced texturing directly translates into superior device stability under prolonged illumination, thermal, and electrical stress. Notably, strongly textured films display significantly reduced light-induced halide segregation compared to controls (Fig. 10c and d). When integrated into PST devices, these optimally textured films demonstrated excellent thermal operational stability, maintaining 80% of their initial efficiency (*T*_80_) after 800 hours of continuous maximum power point operation at 50 °C (Fig. 10e). In addition to improved texturing and crystallinity, enhanced grain size have also been shown to play a crucial role in stabilizing WBG perovskites.^61^ Hu *et al.* demonstrated that perovskite films with larger grains exhibit reduced halide migration and suppressed light-induced phase segregation, leading to improved photo-stability and device performance under continuous illumination.^128^

These studies underscore how controlling facet orientation as well as enhancing crystallinity and texturing *via* crystallization engineering strategies not only mitigates compositional heterogeneities and defect formation, as previously discussed, but also represent a pivotal approach to enhance the intrinsic stability of WBG PSCs.

## 6 Nanoscale impurities

WBG PSCs, particularly those comprising mixed cation (FA^+^, Cs^+^, MA^+^) and mixed halide (I^−^, Br^−^) configurations, inherently host a variety of nanoscale defects. These defects encompass atomic-scale imperfections such as vacancies and interstitials, crystallographically distinct secondary phases, and unreacted precursor precipitates arising during solution processing. Unlike lower-bandgap compositions, with more tolerance for defects,^129^ in WBG perovskites these sites not only impair device performance but also significantly compromise the photostability of the material. Indeed, nanoscale impurities are seeds sites for degradation pathways, highlighting the intrinsic instability introduced by compositional or structural imperfections^53,130,131^

**Fig. 11:**
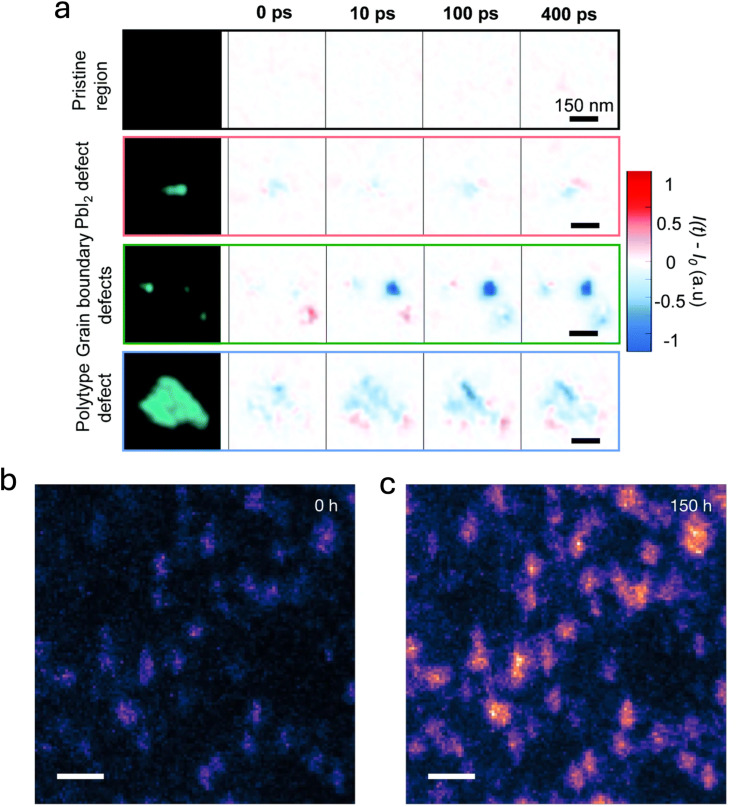
(a) Static PEEM images (left column) of FA_0.78_MA_0.17_Cs_0.05_Pb(I_0.83_Br_0.17_)_3_ films, showing a pristine region without defects and three representative types of defect clusters. Subsequent columns display TR-PEEM intensity variations, plotted as (*I*(*t*) − *I*_0_), at pump–probe delays of 0 ps, 10 ps, 100 ps, and 400 ps. Reproduced from ref. 130 with permission from the Royal Society of Chemistry, Copyright 2021. (b) and (c) Spatially resolved PEEM intensity maps at sub-bandgap energy (*E* − *E*_F_ = −0.83 ± 0.15 eV) recorded on the same FA_0.78_MA_0.17_Cs_0.05_Pb(I_0.83_Br_0.17_)_3_ composition after (b) 0 h and (c) 150 h of *in situ* solar-equivalent illumination. Reprinted from Macpherson *et al.*^131^ Copyright 2022, Springer Nature.

Recent studies have categorized and elucidated the impact of these nanoscale defects on device performance and stability. Kosar *et al.* utilized advanced characterization methods, notably time-resolved photoemission electron microscopy (TR-PEEM), to identify and assess defect clusters in triple-cation mixed-halide perovskites.^130^ Specifically, the authors excited carriers near the perovskite's band edge using near-infrared pump pulses and subsequently imaged the transient occupancy of mid-gap defect states *via* delayed 4.65 eV ultraviolet probe pulses; representative results are illustrated in Fig. 11a. Their investigation revealed that grain-boundary defect clusters, typically a few tens of nanometers in size and likely originating from compositional inhomogeneities, exhibited pronounced variations in photoemission intensity. These observations indicated that such grain-boundary defects serve as significant non-radiative recombination centers, adversely affecting device performance. Conversely, PbI_2_-rich clusters, resulting from incomplete reactions in the precursor solution,^132^ were found to be relatively benign electronically. Such clusters appeared capable of passivating surface defects when present in small quantities, aligning with prior studies suggesting that a controlled excess of PbI_2_ can enhance overall device performance.^133^ Intermediate in severity are hexagonal polytype (δ-phase) inclusions of FAPbI_3_, which reach sizes of a few hundred nanometers. Individually, these polytype inclusions contribute only moderately to non-radiative recombination; however, their collective impact become significantly detrimental when present in higher densities.

**Fig. 12:**
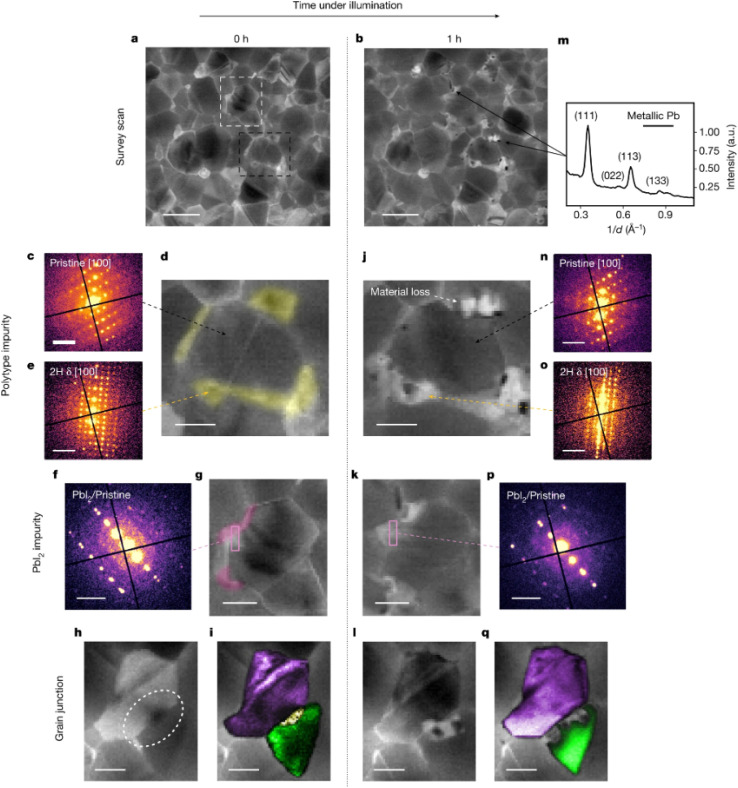
Light-induced degradation in Cs_0.05_FA_0.78_MA_0.17_Pb(I_0.83_Br_0.17_)_3_ thin films occurs at phase impurity sites, as revealed by structural changes tracked post-illumination. (a and b) Diffraction sum images from SED measurements showing the same region of a Cs_0.05_FA_0.78_MA_0.17_Pb(I_0.83_Br_0.17_)_3_ film before (a) and after (b) 1 hour of solar-equivalent illumination *in vacuo* (<10^−6^ mbar). Illumination-induced changes are visible in selected areas. (c) Diffraction pattern indexed to the^100^ zone axis of a tetragonal perovskite (space group *P*4/*mbm*) extracted from the grain indicated by the black arrow in (d). (d) Diffraction sum image from SED data highlighting the region of interest (dashed black box in a) prior to illumination. 2H hexagonal regions adjacent to the grain are marked in yellow. (e) Diffraction pattern from the yellow-highlighted region in (d), indexed to the^100^ zone axis of a 2H hexagonal perovskite. (f) Diffraction pattern from the area indicated in (g), showing a pristine perovskite grain with an epitaxially aligned PbI_2_ grain boundary. Overlapping diffraction spots correspond to perovskite (−333) and PbI_2_ (−330). (g) Diffraction sum image (dashed white box region of a) depicting a pristine perovskite grain encircled by epitaxially aligned PbI_2_ phase impurities (pink). (h) Diffraction sum image of a grain junction. The white dashed ellipse highlights variations in diffraction contrast across the grain. (i) VDF image overlaid on the diffraction sum image from (h), showing a phase impurity (yellow) located at the interface between two grains (purple and green). (j–l) Diffraction sum images from the same regions shown in (d), (g), and (h), respectively, following 1 hour of solar-equivalent illumination. (m) Azimuthally integrated diffraction pattern averaged over several metallic Pb precipitates. (n) Diffraction pattern from a pristine perovskite grain (black arrow in j) post-illumination. (o) Diffraction pattern from a 2H hexagonal impurity phase after illumination. (p) Diffraction pattern from the region in (k) showing both pristine perovskite and epitaxially aligned PbI_2_ after illumination. (q) VDF image overlaid on the diffraction sum image from (l), showing structural changes in the illuminated film. Scale bars: 300 nm (a and b), 0.5 Å^−1^ (c, e, f and n–p), 100 nm (d, g–k, l and q). Reprinted from Macpherson *et al.*^131^ Copyright 2022, Springer Nature.

Further studies have directly connected nanoscale impurities to specific degradation pathways in perovskite solar cells. Macpherson *et al.* explored this link using PEEM mapping.^131^ Through PEEM imaging of sub-bandgap states before and after 150 hours of illumination equivalent to sunlight exposure (Fig. 11b and c), the authors observed that the spatial distribution of trap clusters remain largely unchanged. Regions initially identified as defective exhibit a pronounced increase in sub-bandgap photoemission intensity following illumination, unlike neighboring areas that initially appeared defect-free and remained relatively stable. These findings provided clear evidence that nanoscale defects serve as initial sites for photochemical degradation in formamidinium-rich perovskite absorbers. To further elucidate how these nanoscale defects initiate degradation, the authors performed low-dose scanning electron diffraction (SED) on areas featuring distinct defect clusters before and after illumination (Fig. 12). Initially, the studied regions contained pristine tetragonal perovskite grains (Fig. 12c), along with phase impurities such as nanoscale inclusions of hexagonal polytypes (2H, δ-phase; Fig. 12e) and PbI_2_ (Fig. 12f). Additional phase impurities were located at grain boundaries. After prolonged illumination, significant structural degradation predominantly occurred at impurity sites. These degradation events include the formation of metallic lead precipitates (Fig. 12m) and noticeable areas of material loss (Fig. 12j). The pristine perovskite regions maintained their structural integrity, experiencing only minimal lattice reorientation (Fig. 12n). Conversely, regions containing impurities underwent extensive structural transformations, resulting in the emergence of new structural defects such as vacancies, the formation of PbI_2_, additional hexagonal polytypes (4H), and the nucleation of metallic lead. At grain junctions, impurities further decomposed into amorphous phases, as suggested by the loss of diffraction contrast (Fig. 12l and k). These amorphous phases subsequently expanded into adjacent pristine areas, causing pronounced material loss and the formation of pinholes.

**Fig. 13:**
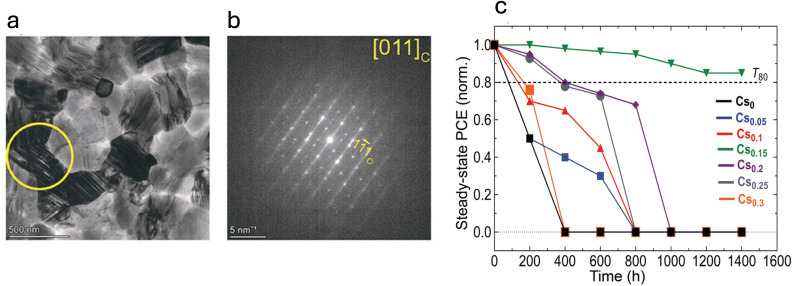
(a) Bright-field (BF) micrograph of a FAPbI_3_ film showing a stacking-fault (SF) domain, with the objective aperture region indicated by the yellow circle. (b) Selected-area electron diffraction (SAED) pattern of the same SF domain, oriented near the [011]_C_ zone axis. (c) Normalized steady-state PCE evolution (averaged over six sub-cells) for encapsulated Cs_*x*_FA_1−*x*_PbI_3_ perovskite solar cells aged under continuous simulated sunlight at 35 °C in a N_2_ environment. Reproduced from Othman *et al.*^53^ with permission from the Royal Society of Chemistry, Copyright 2024.

Another critical type of structural defect in mixed-cation mixed-halide perovskites is stacking faults (SFs). These planar defects, characterized by disruptions in the regular stacking order of atomic planes (Fig. 13a and b), emerge during crystallization and may be a consequence of polymorphism. Their formation is potentially linked to A-site cation inhomogeneities, which introduce localized structural instabilities within the perovskite lattice.^53^ As a result, SFs are formed and act as charge trapping centers, impeding efficient charge transport and ultimately limiting device performance.^53,134,135^ Their role in operational stability was first elucidated by Othman *et al.*, who established a direct correlation between their occurrence and the Cs^+^/FA^+^ molar ratio in the perovskite composition.^53^ Their study demonstrated that films with a higher density of SFs exhibit poorer stability, and notably provided direct imaging evidence showing that degradation initiates and propagates along the length of these stacking faults. Optimizing the cesium content to 15% molar significantly suppressed their formation, minimizing SF occurrence and leading to the most stable devices, as depicted in Fig. 13c.

Given the diverse nature of nanoscale defects identified in mixed halide-cation perovskite solar cells, their effective mitigation necessitates a multidirectional approach. A strategy which has been attempted to pursue this goal involves the complete elimination of Br ions from perovskite compositions, thus forming single-halide mixed-cation systems. This was demonstrated to significantly suppress the formation of hexagonal δ-phase polytypes,^64,131^ enhancing both the structural and photostability of the absorber. Alternatively, structural engineering approaches have also been pursued. For instance, inducing controlled octahedral tilting within the perovskite lattice has shown potential in enhancing stability.^131^ An illustrative case is the incorporation of cyanate pseudohalide (OCN^−^) ions into WBG perovskites, where induced octahedral tilting correlated with notable improvements in operational stability.^83,136^ Additionally, defect-specific passivation strategies, such as controlled exposure to oxygen, have proven selectively effective.^130,131,137^ Oxygen passivation, for instance, efficiently reduces non-radiative recombination at grain boundary defects; however, it exhibits limited effectiveness—or even promotes detrimental chemical reactions—when interacting with hexagonal polytypes or PbI_2_ inclusions.^131^ Complementing these strategies, targeted chemical additives designed for bulk defect passivation, compositional engineering *via* strategic A-and X-site alloying, and incorporation of functional organic molecules provide additional avenues toward simultaneously improving optoelectronic quality and enhancing the overall stability of WBG perovskite solar cells^132,138,139^

## 7 Conclusion and future perspectives

WBG perovskite absorbers, offering suitable bandgaps for PST architectures and the potential for high open-circuit voltages, still suffer from poor operational stability, hindering their broader technological deployment. This review aimed to dissect the fundamental origins of instability in WBG absorbers, including mixed-cation mixed-halide compositions, as well as fully inorganic systems such as CsPbI_3_, with a specific focus on bulk-related degradation mechanisms. Four primary contributors to instability have been highlighted in this review: (i) compositional heterogeneities, both in halide and A-site cation distributions, in mixed-cation mixed-halide formulations; (ii) crystallization-governed structural disorder, which dictates the spatial distribution of elements within the film and strongly affects crystallinity and texturing; (iii) nanoscale impurities, including PbI_2_ domains, stacking faults, grain-boundary trap clusters and hexagonal phase inclusions, which act as non-radiative recombination centres and preferential sites for light- and heat-induced degradation; and (iv) intrinsic phase instability in fully inorganic WBG perovskites, causing the black-to-yellow phase transition in CsPbI_3_.

To mitigate these issues, a wide range of strategies have been proposed and explored. Achieving compositional homogeneity has emerged as a key priority for hybrid mixed-cation mixed-halide absorbers. Where compositional engineering alone, such as reducing the bromide content or optimising the FA^+^/Cs^+^ ratio, proves insufficient, additional control must be exerted during film formation. This includes adjusting deposition routes (*e.g.* replacing antisolvent quenching with gas or vacuum quenching, or adopting multi-step processes that improve precursor mixing), and incorporating functional additives that coordinate with Pb, regulate nucleation, and passivate interfacial and grain-boundary defects. Improving crystallinity and promoting favourable texturing, particularly through facet control, further suppresses defect formation and enhances resistance to external stressors. For inorganic WBG absorbers, whose simpler A- and X-site chemistry helps avoid halide and cation segregation, the dominant challenge becomes stabilising the perovskite phase itself, for example *via* intermediate-phase engineering, compositional tuning and strain management. Complementary to these approaches, defect-targeted passivation strategies offer promising pathways to mitigate trap-induced recombination and limit ion migration. Table 1 provides an overview of the main instability types identified in this work, outlining their physical origins, their impact on device performance and stability, and the corresponding mitigation strategies discussed in this review.

**Table 1:** Summary of intrinsic bulk instabilities of WBG perovskites, their origins, impact on device performance and stability, and representative mitigation strategies

Instability type/defect	Origin (physical/Chemical cause)	Impact on device performance & stability	Potential mitigation strategies	Ref.
Halide compositional heterogeneity (I^−^/Br^−^)	• Different precursor solubility and crystallization kinetics (Br precipitates early)	• Bandgap fluctuations, increased Urbach energy	• Reduce Br content (<20% Br)	37, 47, 62, 64, 79 and 83
	• Antisolvent-assisted growth → Br-rich top surface	• Carrier traps at boundaries between I-rich and Br-rich domains	• Cl-alloying to form Cl-rich intermediate phases	
		• Accelerated light-induced halide segregation	• Pseudohalides (*e.g.* SCN^−^, OCN^−^) to equalize halide incorporation	
			• Additives regulating nucleation (*e.g.* MACl, ABF)	
			• SAM-modified buried interfaces	
A-site cation heterogeneity (FA^+^/Cs^+^ segregation)	• Early Cs^+^ precipitation due to lower solubility	• Local band misalignment, QFLS loss	• Additives coordinating Pb to regulate Cs/FA crystallization	47, 48, 54 and 76
	• Stronger Cs–PbI_3_ interaction → Cs-rich early nuclei	• Increased contact resistance	• Use δ-phase intermediates to enhance Cs distribution	
	• Crystallization-rate mismatch between FA and Cs	• Seed regions for phase impurities and structural defects	• Optimize Cs content to avoid over-/under-doping	
Nanoscale impurity phases (PbI_2_ domains, δ-phase inclusions)	• Incomplete precursor reaction	• Moderate to severe nonradiative recombination	• Improve precursor stoichiometry and mixing	47, 53, 130 and 131
	• Polymorphism in mixed halide–cation systems	• Defects act as nucleation centers for light-induced degradation	• Suppress δ-phase formation *via* pseudohalides and FA/Cs tuning	
	• Poor control during rapid crystallization	• Growth of metallic Pb and amorphous phases under illumination	• Use additives forming uniform nucleation (*e.g.* ABF, OCN^−^)	
Stacking faults/planar defects	• Polymorphism and A-site inhomogeneity	• Trap-assisted recombination	• Control FA/Cs ratio (≈15% Cs gives minimum SF density)	53, 134 and 135
	• Local strain fields promoting faulted layers	• Preferential degradation along the fault planes	• Promote uniform A-site compositional mixing	
		• Reduced photostability	• Improve texturing and crystallinity	
Grain-boundary trap clusters	• Nonuniform crystallization	• Severe nonradiative recombination	• Additives improving nucleation uniformity (*e.g.* ABF, PSP)	64, 130 and 131
	• Local halide/cation fluctuations	• Ion migration pathways	• Gas/vacuum quenching to enlarge grains and improve texture	
	• Structural impurities accumulating at grain boundaries	• Early degradation onset under illumination	• Oxygen passivation (defect-specific)	
Facet-dependent instability	• Uneven exposure of (100) *vs.* (111) facets	• (100) facets degrade faster *via* hydration and beam damage	• Induce preferential (111) faceting using texture-control additives (*e.g.* TOPO)	125, 126, 128 and 140
	• Intrinsic facet-specific water adsorption and ion migration barriers	• (111) facets more stable under heat/light	• Vacuum/gas-quenching to strengthen preferred orientation	
Phase instability in CsPbI_3_ (α/β/γ → δ)	• Low tolerance factor → octahedral tilting	• Transition to non-perovskite yellow δ-phase	• DMAI/DMA^+^ intermediate stabilization	28, 29, 87, 88, 93 and 94
	• Soft lattice and anharmonic phonons	• Severe optoelectronic losses	• Additives (*e.g.* PVP, organic cations) and/or 2D capping layers	
	• Surface iodide vacancies trigger δ-phase nucleation	• Moisture accelerates yellow-phase growth	• X-site alloying (*e.g.* Br, Cl alloying)	
			• Strain engineering	

Across the literature surveyed here, several recurring themes point toward promising future directions for achieving the long-term operational stability required for PST integration: (i) deeper understanding and control of crystallization dynamics to suppress heterogeneity at the earliest stages of film formation; (ii) systematic suppression of nanoscale impurity phases, supported by advanced nanoscale characterization; (iii) intentional facet and texture engineering to stabilise the absorber under light and heat; (iv) strategies that promote homogeneous ion incorporation in mixed compositions without sacrificing the desired bandgap; and (v) coupled bulk-interface stability concepts that address the interconnected nature of recombination sites and ionic pathways. Addressing these aspects in WBG absorbers will be critical to unlocking their full potential and enabling the long-term operational stability required for next-generation tandem PVs.

## Author contributions

C. O. conceived and led the review; curated the literature and data; carried out the investigation and visualization; developed the methodology; and wrote the original draft. M. O. contributed to conceptualization, methodology, investigation, formal analysis, validation, and supervision, and assisted with drafting; he also participated in review and editing. C. M. W. and A. H.-W. contributed to conceptualization and methodology, provided supervision and resources, shared responsibility for project administration and funding acquisition, and contributed to data curation and manuscript revision. C. B. contributed to funding acquisition and provided oversight, resources, and administrative support, and participated in manuscript review and editing. All authors discussed the work, revised the manuscript, and approved the final version.

## Conflicts of interest

The authors declare no conflicts of interest.

## Supplementary Material

EL-002-D5EL00199D-s001

## Data Availability

This article is a review and does not report original experimental data. Accordingly, no new datasets, software, or codes were generated as part of this work. All information and interpretations are derived from previously published studies, which are properly cited in the text. Supplementary information (SI) is available. See DOI: https://doi.org/10.1039/d5el00199d.
