# Supplementary material for: Unraveling bulk degradation mechanisms of wide-bandgap perovskite absorbers for tandem applications
Source: EES Solar. 2026 Feb 26;2(2):293–314. doi: 10.1039/d5el00199d (PMC12968988; doi:10.1039/d5el00199d)
Supplement: EL-002-D5EL00199D-s001 [file EL-002-D5EL00199D-s001.pdf]

Supplementary Information (SI) for EES Solar.
This journal is © The Royal Society of Chemistry 2026

1 Supporting information

Bandgap (eV)	Encaps. (yes/no)	RH (%)	Temp. (°C)	Cond. (MPP/OC)	Active area (cm^2)	Masked? (yes/no)	T80 (h)	PCE@RT (%)	Author	Year	Ref.
1.62	yes	–	90	MPP	0.0625	yes	24467	24.58	Li *et al.*	2025	[1]
1.65	yes	–	55	OC	0.08	–	4000	23.20	Yang *et al.*	2025	[2]
1.60	yes	60%	85	OC	0.25	yes	6400	22.60	Lin *et al.*	2024	[3]
1.66	no	N_2 atm.	65	MPP	0.0737	yes	994	24.53	Wang *et al.*	2024	[4]
1.60	no	N_2 atm.	85	MPP	0.25	yes	5000	25.10	Wang *et al.*	2024	[5]
1.83	no	N_2 atm.	50	MPP	0.0628	yes	2222	18.96	Zhang *et al.*	2024	[6]
1.63	no	N_2 atm.	85	MPP	0.10	yes	765	21.10	Ren *et al.*	2024	[7]
1.65	yes	–	65	MPP	1.00	yes	8521	23.48	Li *et al.*	2023	[8]
1.67	no	N_2 atm.	55	MPP	0.056	yes	1866	22.28	Hang *et al.*	2023	[9]
1.72	yes	50%	85	MPP	0.16	–	5333	17.40	Zhao *et al.*	2022	[10]
1.76	yes	50%	85	OC	0.0919	yes	490	16.70	McMeekin *et al.*	2022	[11]
1.75	no	N_2 atm.	65	MPP	0.112	yes	4400	20.00	Jiang *et al.*	2022	[12]
1.66	yes	50%	85	OC	0.0919	yes	4800	20.10	Lin *et al.*	2020	[13]
1.60	no	N_2 atm.	85	MPP	0.104	yes	2500	22.02	Wu *et al.*	2020	[14]
1.67	no	N_2 atm.	65	MPP	0.06	yes	5000	20.42	Xu *et al.*	2020	[15]
1.60	yes	60%	65	MPP	–	–	7500	21.20	Yang *et al.*	2019	[16]
1.60	yes	45%	75	OC	0.0919	yes	1440	18.70	Bai *et al.*	2019	[17]

Table S 1: Summary of stability tests for wide-bandgap perovskite solar cells. N_2 atm. = nitrogen atmosphere; PCE@RT = power conversion efficiency at room temperature. *Cond.* refers to the measurement conditions used for stability testing: MPP = maximum power point tracking; OC = open circuit. When the actual active area used for stability testing was not explicitly reported, the masked area used for efficiency measurements is shown in the ”Active area” column.

References

[1] Qing Li et al. “Graphene-polymer reinforcement of perovskite lattices for durable solar cells”. In: *Science* 387.6738 (2025), pp. 1069–1077.

[2] Guang Yang et al. “Reductive cation for scalable wide-bandgap perovskite solar cells in ambient air”. In: *Nature Sustainability* (2025), pp. 1–8.

[3] Yen-Hung Lin et al. “Bandgap-universal passivation enables stable perovskite solar cells with low photovoltage loss”. In: *Science* 384.6697 (2024), pp. 767–775.

[4] Zhenhan Wang et al. “Regulation of wide bandgap perovskite by rubidium thiocyanate for efficient silicon/perovskite tandem solar cells”. In: *Advanced Materials* 36.50 (2024), p. 2407681.

[5] Wei-Ting Wang et al. “Water-and heat-activated dynamic passivation for perovskite photovoltaics”. In: *Nature* 632.8024 (2024), pp. 294–300.

[6] Zhichao Zhang et al. “Suppression of phase segregation in wide-bandgap perovskites with thiocyanate ions for perovskite/organic tandems with 25.06% efficiency”. In: *Nature Energy* 9.5 (2024), pp. 592–601.

[7] Xiaoxue Ren et al. “Mobile iodides capture for highly photolysis-and reverse-bias-stable perovskite solar cells”. In: *Nature Materials* 23.6 (2024), pp. 810–817.

[8] Lin Li et al. “Buried-interface engineering enables efficient and 1960-hour ISOS-L-2I stable inverted perovskite solar cells”. In: *Advanced Materials* 36.13 (2024), p. 2303869.

[9] Pengjie Hang et al. “Highly efficient and stable wide-bandgap perovskite solar cells via strain management”. In: *Advanced Functional Materials* 33.11 (2023), p. 2214381.

[10] Xiaoming Zhao et al. “Accelerated aging of all-inorganic, interface-stabilized perovskite solar cells”. In: *Science* 377.6603 (2022), pp. 307–310.

[11] David P McMeekin et al. “Intermediate-phase engineering via dimethylammonium cation additive for stable perovskite solar cells”. In: *Nature Materials* 22.1 (2023), pp. 73–83.

[12] Qi Jiang et al. “Compositional texture engineering for highly stable wide-bandgap perovskite solar cells”. In: *Science* 378.6626 (2022), pp. 1295–1300.

[13] Yen-Hung Lin et al. “A piperidinium salt stabilizes efficient metal-halide perovskite solar cells”. In: *Science* 369.6499 (2020), pp. 96–102.

[14] Shengfan Wu et al. “2D metal–organic framework for stable perovskite solar cells with minimized lead leakage”. In: *Nature Nanotechnology* 15.11 (2020), pp. 934–940.

[15] Jixian Xu et al. “Triple-halide wide–band gap perovskites with suppressed phase segregation for efficient tandems”. In: *Science* 367.6482 (2020), pp. 1097–1104.

[16] Shuang Yang et al. “Stabilizing halide perovskite surfaces for solar cell operation with wide-bandgap lead oxysalts”. In: *Science* 365.6452 (2019), pp. 473–478.

[17] Sai Bai et al. “Planar perovskite solar cells with long-term stability using ionic liquid additives”. In: *Nature* 571.7764 (2019), pp. 245–250.
